# Multi-Time Resolution Ensemble LSTMs for Enhanced Feature Extraction in High-Rate Time Series

**DOI:** 10.3390/s21061954

**Published:** 2021-03-10

**Authors:** Vahid Barzegar, Simon Laflamme, Chao Hu, Jacob Dodson

**Affiliations:** 1 Department of Civil, Construction, and Environmental Engineering, Iowa State University, 813 Bissell Road, Ames, IA 50011, USA; laflamme@iastate.edu; 2 Department of Electrical and Computer Engineering, Iowa State University, Ames, IA 50011, USA; chaohu@iastate.edu; 3 Department of Mechanical Engineering, Iowa State University, Ames, IA 50011, USA; 4 Air Force Research Laboratory, Munitions Directorate, Fuzes Branch, Eglin Air Force Base, FL 32542, USA; jacob.dodson.2@us.af.mil

**Keywords:** sensor measurement, deep learning, nonlinear, recurrent neural networks, long short-term memory, non-stationary, time series, high-rate, prediction

## Abstract

Systems experiencing high-rate dynamic events, termed high-rate systems, typically undergo accelerations of amplitudes higher than 100 g-force in less than 10 ms. Examples include adaptive airbag deployment systems, hypersonic vehicles, and active blast mitigation systems. Given their critical functions, accurate and fast modeling tools are necessary for ensuring the target performance. However, the unique characteristics of these systems, which consist of (1) large uncertainties in the external loads, (2) high levels of non-stationarities and heavy disturbances, and (3) unmodeled dynamics generated from changes in system configurations, in combination with the fast-changing environments, limit the applicability of physical modeling tools. In this paper, a deep learning algorithm is used to model high-rate systems and predict their response measurements. It consists of an ensemble of short-sequence long short-term memory (LSTM) cells which are concurrently trained. To empower multi-step ahead predictions, a multi-rate sampler is designed to individually select the input space of each LSTM cell based on local dynamics extracted using the embedding theorem. The proposed algorithm is validated on experimental data obtained from a high-rate system. Results showed that the use of the multi-rate sampler yields better feature extraction from non-stationary time series compared with a more heuristic method, resulting in significant improvement in step ahead prediction accuracy and horizon. The lean and efficient architecture of the algorithm results in an average computing time of 25 μs, which is below the maximum prediction horizon, therefore demonstrating the algorithm’s promise in real-time high-rate applications.

## 1. Introduction

High-rate dynamic systems are engineering systems subjected to high-amplitude dynamic events, often higher than 100 g${}_{n}$ (g-force), and over very short durations, typically under 100 ms. Enabling closed-loop feedback capabilities for high-rate systems, such as hypersonic vehicles, advanced weaponry, and airbag deployment systems, is driven by the need to ensure continuous operations and safety. Such capabilities require high-rate system identification and state estimation, defined as high-rate structural health monitoring (HRSHM), through algorithms capable of sub-millisecond decisions using sensor measurements [1]. However, the development of HRSHM algorithms is a difficult task, because the dynamics of high-rate systems is uniquely characterized by (1) large uncertainties in the external loads, (2) high levels of non-stationarity and heavy disturbances, and (3) unmodeled dynamics generated from changes in the system configurations [2].

There have been recent research efforts in constructing algorithms for HRSHM by integrating some levels of physical knowledge about the system of interest, in particular on an experimental setup constructed to reproduce high-rate dynamics that consists, among other features, of a cantilever beam equipped with a moving cart acting as a sliding boundary condition [3]. Using these experimental data acquired from an accelerometer located under the beam, Joyce et al. [3] presented a sliding mode observer to track the position of the moving cart through the online identification of the fundamental frequency. Downey et al. [4] proposed to track the cart location using a real-time model-matching approach of the fundamental frequency extracted using a Fourier transform, and experimentally demonstrated the promise of the algorithm on the test setup. Yan et al. [5] developed a model reference adaptive system algorithm consisting of a sliding mode observer used in updating a reduced-order physical representation of the dynamic system, and numerically showed the sub-millisecond capabilities in tracking the cart location.

While these algorithms showed great promise, the dominating dynamics of the experimental setup was relatively simple, where the position of the cart could be mapped linearly to the beam’s first fundamental frequency. However, high-rate systems in field applications are rarely that simple, and experimental data is difficult and expensive to acquire. It follows that one must assume low levels of physical knowledge in designing HRSHM algorithms. A solution is to leverage data-based techniques, which may also be beneficial in increasing the computational efficiency of the algorithms [6]. To cope with the challenges of limited training data, high non-stationarities, and high uncertainties, a desirable HRSHM algorithm is one capable of adaptive behavior, ideally in real time [2].

Among data-driven algorithms, neural network-based models have been successfully applied to model complex nonlinear dynamic systems across many fields [7,8], including electricity demand prediction [9], biology [10], autonomous vehicles [11,12], and structural health monitoring [13,14,15]. Among these models, recurrent neural networks (RNNs) are of interest to the HRSHM problem due to their temporal dynamic characteristics, where they are capable of recognizing sequential patterns by selectively processing information [16]. Long short-term memory (LSTM) networks are a specialized type of RNN capable of capturing long-term temporal dependencies [17], and have thus been successfully applied to modeling time series measurements [18,19,20,21], including modeling multivariate time series [22,23] and reconstructing attractors [24]. Some work on LSTM focused on the problem of prediction for non-stationary systems. For instance, Guen and Thome [19] introduced a new loss function based on both temporal errors and distortion of future predicted trajectories to enforce learning in a highly non-stationary environment. Cui et al. [20] used multi-layer bidirectional LSTMs to better capture spatial features of traffic data for large scale traffic network prediction. Hua et al. [25] introduced random connections in the LSTM architecture to better cope with non-stationarity in the problem of traffic and mobility prediction. Yeo and Melnyk [26] introduced a probabilistic framework for predicting noisy time series with RNNs.

The architectures proposed in the existing methods necessitate the use of large-sized networks, which require relatively long computing times. This limits the application of these methods to HRSHM. It is also noted that leveraging data-based algorithms for HRSHM requires a certain level of on-the-edge learning due to the complex dynamics under consideration and the limited availability of training data. The adaptive algorithm must be capable of rapid convergence to ensure adequate performance while guaranteeing fast computing to empower real-time applicability. The introductory paper on HRSHM [2] discussed the important conflict between convergence speed and computing time, where convergence speed generally increases with the algorithm’s complexity while computing time decreases.

A viable approach to improving both convergence speed and computing time is to incorporate physical knowledge into data-based architectures, a process also known as physics-informed machine learning [27]. Such knowledge integration can be done, for example, in the form of accompanying logic rules [28], algebraic equations [29], and mechanistic models [30,31]. By incorporating physical knowledge, the algorithm can be designed to converge more efficiently, therefore preserving a leaner or less complex architecture, and thus favoring faster computing. Of interest, the authors have proposed in [32] a purely on-the-edge learning wavelet neural network that exhibited good convergence properties by varying its input space as a function of the extracted local dynamic characteristics of the time series. This information on the time series data structure was based on Takens’ embedding theorem [33] which constituted the physical information fed to the wavelet network. The objective was to demonstrate that a machine learning algorithm could learn a non-stationary representation without pre-training. While successful, the architecture of the algorithm itself would not converge because of the constantly changing input space, and the computing time required to extract the local dynamic characteristics was too long for HRSHM applications.

The objective of this paper is to investigate the performance of a physics-informed deep learning method in predicting sensor measurements enabling HRSHM, inspired by the authors’ prior work in [32]. Instead of a time-varying input space, the algorithm uses an ensemble of RNNs, each using a different delay vector to represent distinct local data structures in the dynamics. The novelty lies in the incorporation of physics in the algorithm, which stems from the pre-analysis of available training data to extract the appropriate delay vector characteristics for each RNN after the identification of the time series data structure through principal component analysis (PCA). This allows the network to extract local features in the time series in order to provide improved multi-step ahead prediction performance capabilities. The physics-informed deep learning method shows improved performance over a conventional grid search method. Short-sequence LSTMs are used to improve computing speed, and transfer learning [34] is used to adapt the representation to the target domain.

The rest of the paper is organized as follows. Section 2 provides the algorithm used for prediction. Section 3 describes the proposed input space construction method. Section 4 presents the validation method including the experimental test used to collect high-rate data and performance metrics. Section 5 and Section 6 present the validation results and a discussion about their implications, respectively. Section 7 concludes the paper.

## 2. Deep Learning Architecture

**Figure 1:**
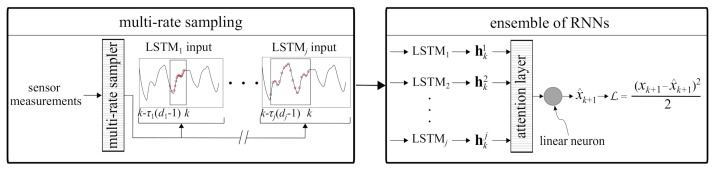
Deep learning architecture: ensemble of RNNs for one-step ahead prediction.

This section presents the background on the algorithm used to conduct step ahead predictions of non-stationary time series measurements **x** while sensor measurements are being acquired. The algorithm, shown in Figure 1, consists of (1) a multi-rate sampler, shown in Figure 1 (left), and (2) an ensemble of *j* LSTM cells arranged in parallel, joined through an attention layer, to conduct the prediction $\hat{x}$, shown in Figure 1 (right) as making a one-step ahead prediction at step *k*, ${\hat{x}}_{k+1}$. Short-sequence LSTMs are used to accelerate computing. A particularity of the algorithm is that the multi-rate sampler is used on part of the acquired sensor measurements $x=[\begin{array}{cccc} x_{1} & x_{2} & \cdots & x_{k} \end{array}]$ to represent unique dynamic characteristics. More precisely, it samples a different sequence for each LSTM *i* (i=1,2,⋯,j) using a different time delay $\tau_{i}$ embedded in a vector of dimension $d_{i}$ with the delay vector $x_{k}^{i}$ written

(1) $$\begin{array}{ccccc} x_{k}^{i}=[x_{k+1-d_{i}\tau_{i}} & x_{k+1-\left(d_{i}-1\right)\tau_{i}} & \cdots & x_{k+1-2\tau_{i}} & x_{k+1-\tau_{i}}] \end{array}$$

where τ is a positive integer. Note that in Equation (1), $x_{k}^{i}$ is organized such that each individual one-step ahead prediction ${\hat{x}}_{k+1}^{i}$ is temporally consistent. The choice for τ and *d* in the multi-rate sampler is based on physics and constitutes the novelty of the proposed algorithm. It will be described in the next section.

**Figure 2:**
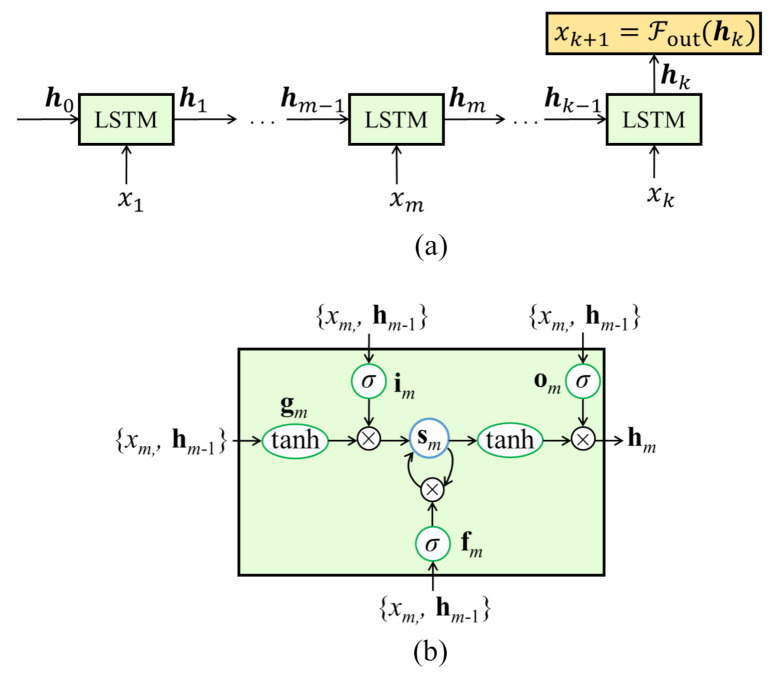
(**a**) Unfolded basic RNN; and (**b**) LSTM cell architecture.

The use of input spaces of different time resolutions allows capturing different local dynamics that can be fed into different LSTM cells to extract multi-resolution dynamics features. The role of the attention layer and linear neuron is to combine these extracted features to model the dynamics of the system. Each individual LSTM cell uses the delay vector to recursively update the hidden state h. Hidden state $h_{k}$ at time step *k* represents a feature vector used in conducting the prediction, with

(2) $$h_{k}=r\left(h_{k-\tau },x_{k}\right)$$

where *r* is the updating function of an LSTM cell, with $h_{0}$ commonly initialized at zero [16]. The recursive update process of an RNN with LSTM cells is illustrated in Figure 2a, and the internal architecture of a single LSTM cell in Figure 2b. LSTMs are defined by the following equations [17]

(3) $$\begin{array}{cc} g_{m} & =\tanh(W_{x}^{g}x_{m}+W_{h}^{g}h_{k-\tau }+b_{g}) \end{array}$$

(4) $$\begin{array}{cc} i_{m} & =\sigma (W_{x}^{i}x_{m}+W_{h}^{i}h_{m-\tau }+b_{i}) \end{array}$$

(5) $$\begin{array}{cc} f_{m} & =\sigma (W_{x}^{f}x_{m}+W_{h}^{f}h_{m-\tau }+b_{f}) \end{array}$$

(6) $$\begin{array}{cc} o_{m} & =\sigma (W_{x}^{o}x_{m}+W_{h}^{o}h_{m-\tau }+b_{o}) \end{array}$$

(7) $$\begin{array}{cc} s_{m} & =f_{m}⊙s_{m-\tau }+i_{m}⊙g_{m} \end{array}$$

(8) $$\begin{array}{cc} h_{m} & =o_{m}⊙\tanh\left(s_{m}\right) \end{array}$$

where tanh(·), and σ(·) represent hyperbolic tangent and the logistic sigmoid functions, respectively, $W_{x}$ and $W_{h}$ the input and output weights, respectively, **b** the bias vector associated with the gate in subscript, and ⊙ an element-wise multiplication. Both weights and biases are shared through all time steps. These LSTM cells use internal gating functions (Equations (3)–(8)) to augment memory capabilities. Three gates, consisting of the input gate i, forget gate f, and output gate o, modulate the flow of information inside the cell by assigning a value in the range of (0,1) to write the input to the internal memory s ($i_{m}⊙g_{m}$ in Equation (7)), reset the memory ($f_{m}⊙s_{m-1}$ in Equation (7)), or read from it (Equation (8)). Gate values close to zero are less relevant for prediction purposes than those with values close to one.

The one-step ahead prediction is based on a linear combination of the features extracted by the LSTM cells in the ensemble. The combination of features is conducted in two steps. First, an attention layer determines dominant features by assigning attention weights $\alpha_{i}\in R$ for i=1,2,⋯,j to the LSTM outputs. Second, a linear neuron combines the scaled features to produce the one-step ahead prediction ${\hat{x}}_{k+1}$. This architecture can also be used to predict multiple steps ahead. The idea is to iteratively execute one-step ahead prediction *q* times to predict a *q*-step trajectory. For the *i*th LSTM cell in the ensemble, the moving window of the multi-rate sampler continues to provide inputs from the measured time series up to $q<\tau_{i}$. For $q>\tau_{i}$, the predicted measurements are appended to the actual measurements to construct the input space.

## 3. Multi-Rate Sampler

This section describes the multi-rate sampler used to incorporate physics into the deep-learning algorithm by selecting an input space for each LSTM based on essential local dynamics. Each individual input space is used in the training of the associated feature extractors. An input with a large time delay is less influenced by the fast-changing dynamics and thus focuses on the slow-changing dynamics. Conversely, an input with a small time delay is more sensitive to fast-changing dynamics. These time delays are determined through a decomposition of the signal into its principal components (PCs) using PCA. The embedding theorem is used to extract physical properties from each principal component in the form of a delay vector, and each delay vector becomes the input space of an associated LSTM cell. Hence, a decomposition of the sensor measurements into *j* principal components will lead to an ensemble of *j* LSTM cells. In this section, the PCA procedure on time series measurements is presented, followed by the approach to constructing the delay vectors and procedure to train the feature extractor based on the delay vectors.

### 3.1. Principal Component Analysis

PCA is used to identify data structure in the time series by decomposing the training sensor measurements into dominating principal components. Consider a one-dimensional zero-mean time series $V=[\begin{array}{cccc} \nu_{1} & \nu_{2} & \cdots & \nu_{n} \end{array}]$. A delayed observation matrix X consisting of *m*-variate delayed vectors of the form $v_{i}=\left[\begin{array}{cccc} \nu_{i} & \nu_{i+1} & \cdots & \nu_{i+(m-1)} \end{array}\right]$ can be constructed in a delay embedding space *m* as follows [35]

(9) $$X=\left[\begin{array}{c} v_{1}\\ v_{2}\\ .\\ .\\ .\\ v_{n-m+1} \end{array}\right]={\left[\begin{array}{ccccc} \nu_{1} & \nu_{2} & \nu_{3} & \ldots & \nu_{m}\\ \nu_{2} & \nu_{3} & \nu_{4} & \ldots & \nu_{m+1}\\ . & . & . & \; & .\\ . & . & . & \; & .\\ . & . & . & \; & .\\ \nu_{n-m+1} & \nu_{n-m+2} & \nu_{n-m+3} & \ldots & \nu_{n} \end{array}\right]}_{(n-m+1)\times m}$$

The principal components of the observation matrix are obtained through the singular value decomposition

(10) $$X=S\Sigma C^{T}$$

where S is the (n−m+1)×(n−m+1) eigenvectors of ${\mathrm{XX}}^{T}$, C is the m×m eigenvectors of $X^{T}X$, and Σ is the associated (n−m+1×m) principal components matrix. The reconstruction of the time series V based on the *i*th singular value is given by

(11) $$V_{i}=S_{\cdot ,i}\Sigma_{i,i}C_{\cdot ,i}^{T}$$

where · denotes all the rows of the matrix.

The decomposed signal is expected to resemble the signal’s frequency components for m→n, yet yielding relevant structures for smaller *m* values [35]. Here, the objective is for the feature extractor to operate in different representative time resolutions while keeping the machine learning architecture lean to maintain fast computing. This is done by keeping *m* small and using the first principal components that reconstruct the original time series with at least 95% accuracy, in terms of mean absolute error, with each additional principal component reconstruction representing a signal that is richer in higher frequency components.

### 3.2. Construction of Input Space

The construction of the input space through the use of different delay vectors is based on Takens’ embedding theorem which states that the phase-space of an autonomous system can be topologically reconstructed using a set of delayed observations from a single state embedded in a delay vector (Equation (1)). This delay vector is said to preserve the essential dynamics of the system, given that τ and *d* are appropriately selected. The theorem has been extended to non-autonomous systems with deterministic forcing [36], state-dependent forcing [37], and stochastic forcing [38].

Here, the approach is to assume that a delay vector exists for each principal component that preserves the essential dynamics of the reconstructed time series, thus constituting an input space rich in information on the dynamics of interest. There exist well-established numerical techniques for selecting proper values of τ and *d*. Here, τ is selected based on the mutual information (MI) test [39], while *d* is selected based on the false nearest neighbors (FNN) test [32]. The MI test is based on information theory. It measures the nonlinear dependence of measurements for different sampling periods and selects a delay value that adds the most information to the sequence. Fraser and Swinney [40] recommend selecting the first local minimum of the MI curve as the optimal time delay, with the subsequent local minima adding unnecessary complexity to the phase-space of the reconstructed system. The FNN test searches for an optimal dimension *d* by evaluating the changes in the ranks of neighboring states along different dimensions, and computes the percentage of false neighbors per dimension. The optimal dimension is one that has the smallest percentage of false neighbors and is typically selected based on an FNN threshold. Here, this threshold is taken as 5%. Hence, for each reconstructed time series from the corresponding principal component, both the MI and FNN tests are conducted to identify optimal values of τ and *d*, which are used in constructing the *i*th delay vector used as the input space for the associated *i*th LSTM (i.e., feature extractor) at any given time step *k*

(12) $$x_{k}^{i}=\left[\begin{array}{cccc} x_{k-\left(d_{i}-1\right)\tau_{i}} & x_{k-\left(d_{i}-2\right)\tau_{i}} & \cdots & x_{k} \end{array}\right]$$

### 3.3. Feature Extractor Training

The proposed HRSHM algorithm assumes that only limited training data, termed source domain, is available and that such data does not represent all of the possible dynamics of the system. The source domain has the learning task of extracting features. Transfer learning [41] is used to transfer knowledge from the source domain to the real-time learning domain, termed target domain.

**Figure 3:**
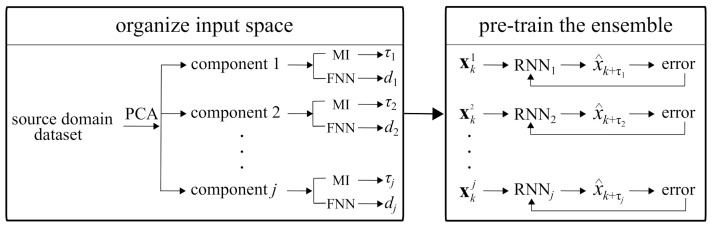
Feature extractors training algorithm.

The algorithm for training the feature extractors is shown in Figure 3. First, *j* principal components are extracted from the source domain via PCA and *j* input spaces **X** constructed using associated delay vector characteristics (τ and *d*). After, an RNN with LSTM cells (as in Figure 2a), with a hidden state *h* of size equal to twice its corresponding embedding dimension *d*, is trained on the original training time series (i.e., pre-decomposition) using a standard sequential back-propagation scheme with the associated loss function

(13) $$L=\frac{{\left(x_{k+\tau }-{\hat{x}}_{k+\tau }\right)}^{2}}{2}$$

where *x* and $\hat{x}$ are the true and estimated values, respectively. It follows that each RNN is constructed to predict the associated τ steps ahead. The LSTM weights are then used as feature extractor weights in the target domain (Figure 1). When assembled, each LSTM cell in the RNN is appropriately delayed to temporally align the estimates, here taken as a single-step ahead, ${\hat{x}}_{k+1}$ (Equation (1)).

## 4. Validation Methodology

The proposed deep learning algorithm is validated on experimental data obtained from a series of drop tower tests. In what follows, the experimental test setup is described, and the performance metrics defined.

### 4.1. Experimental Setup

**Figure 4:**
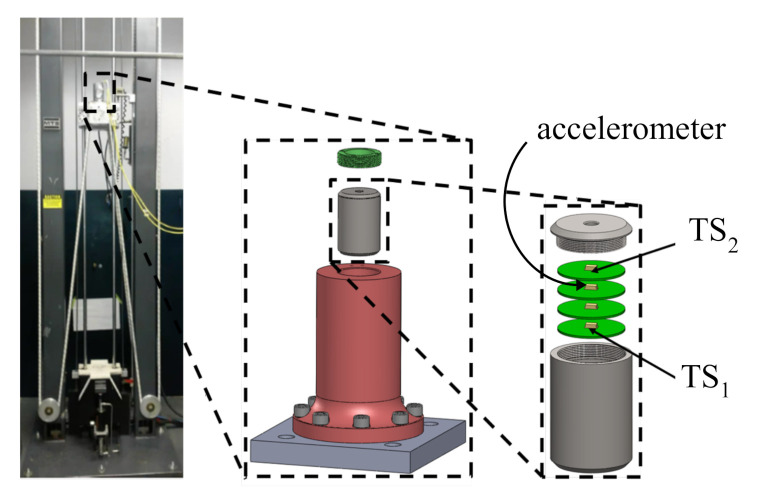
Drop tower experimental setup [32].

The proposed HRSHM algorithm is validated on high-rate dynamic datasets obtained experimentally from accelerated drop tower tests. The experimental configuration is illustrated in Figure 4 and described in detail in [32]. Briefly, four circuit boards, each equipped with an accelerometer capable of measuring up to 120,000 g${}_{n}$ (or 120 kg${}_{n}$), were placed inside a canister with the electronics secured using a potting material. The canister was dropped five consecutive times and deceleration responses of the circuit boards were recorded with a sampling rate of 1 MHz (k=1 μs).

**Figure 5:**
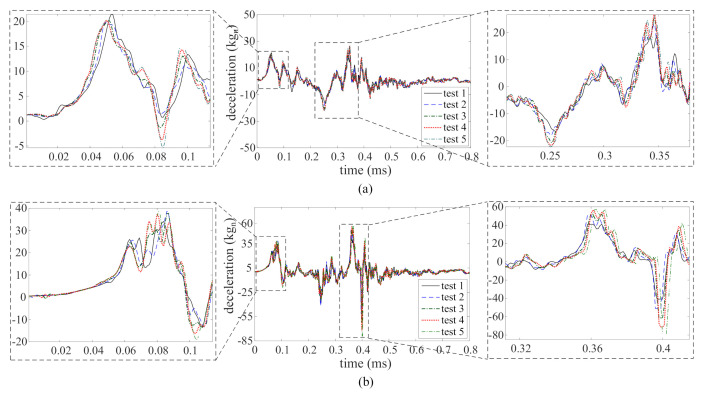
Deceleration time series obtained from five consecutive tests: (**a**) TS${}_{1}$; and (**b**) TS${}_{2}$.

Figure 5a,b plot the recorded time series for TS${}_{1}$ and TS${}_{2}$ obtained from accelerometers 1 and 2, respectively, through five consecutive tests. From these time series plots, one can observe the following high-rate dynamic characteristics: (1) the magnitude of deceleration is high, in the kg${}_{n}$ range; (2) the duration of the excitation occurs during a very short time frame, under 1 ms; (3) the dynamics exhibits high levels of non-stationarities; and (4) the dynamic response is altered after each test, likely attributable to the whipping of cables and/or damage in the potting material housing the electronics.

In this work, the acceleration time series TS${}_{1}$ and TS${}_{2}$ are used for the algorithm validation. In particular, it is assumed that only test 1 from TS${}_{1}$ is available for training (source domain), and is therefore used to construct the input space. The target domain consists of tests 1–5 from TS${}_{2}$, used to conduct real-time prediction while sensor data is being acquired.

### 4.2. Performance Metrics

Four performance metrics are defined to evaluate the proposed algorithm. The first two metrics are the mean absolute error (MAE) and root mean square error (RMSE) of the prediction, defined as

(14) $$\mathrm{MAE}=\frac{1}{n}\sum_{k=1}^{n}\left|x_{k+1}-{\hat{x}}_{k+1}\right|$$

(15) $$\mathrm{RMSE}=\sqrt{\frac{1}{n}\sum_{k=1}^{n}{\left(x_{k+1}-{\hat{x}}_{k+1}\right)}^{2}}$$

where *n* is the number of samples.

The third metric is the naive prediction length, where a naive prediction is defined as the predicted value of a future step being equal to that of the immediate previous step. This behavior manifests itself as a flat horizontal line in a time series plot. To detect a naive prediction, a moving window of length *q* equal to that of the prediction horizon is moved along both the real and predicted time series, and the standard deviations are computed. If the standard deviation of the prediction is arbitrarily less than 50% of the standard deviation of the original time series, the prediction values within the window are reported as a naive. The total length of naive prediction windows, in terms of number of prediction steps, over the length of the time series forms the naive metric.

The fourth metric is based on dynamic time warping (DTW) [42]. DTW searches for local similarities between two sequences (i.e., between the real and predicted time series) by compressing or stretching them. The DTW metric for two sequences A and B is obtained by sliding a window of length *l* over A and B to construct a global distance matrix D, where the local element $D_{k}\left(i,j\right)$ is the distance between the *i*th point in A and *j*th point in B within the *k*th non-overlapping window and takes the following form

(16) $$D_{k}\left(i,j\right)=\left|A(i)-B(j)\right|+\min\left(D_{k}\left(i-1,j-1\right),D_{k}\left(i-1,j\right),D_{k}\left(i,j-1\right)\right)$$

The global distance matrix D is created by appending local matrices diagonally as

(17) $$D={\left[\begin{array}{cccc} D_{1} & 0 & 0 & \cdots \\ 0 & D_{2} & 0 & \cdots \\ 0 & 0 & D_{3} & \cdots \\ \vdots & \vdots & \vdots & \ddots \end{array}\right]}_{n\times n}$$

A warping path is obtained by starting at the first element of the distance matrix D(1,1), and moving to the right, down, or diagonally by following the minimum elements. The DTW value is taken as the sum of the elements on the warping path, where a smaller DTW value indicates greater similarity between the two sequences. The metric is applied here to measure the similarity between the features extracted by the LSTMs and the sensor measurements.

For all four performance metrics, smaller values indicate better performance of the algorithm and are thus desirable. In the numerical study that follows, the HRSHM algorithm that incorporates physical knowledge (“PCA inputs”) is compared against the so-called grid-search (“GS inputs”) method where τ’s are selected based on a visual inspection of the topology of the phase-space of the source domain and *d* optimized to obtain the best performance for one-step ahead predictions. Remark that there are other methods available for selecting the hyper-parameters of a neural network, including Bayesian [43,44] and evolutionary optimization [45,46] techniques. Nevertheless, given the small number of hyper-parameters inherent to our algorithm, the “GS inputs” method is deemed appropriate for comparison. Results are also benchmarked against the one-step ahead prediction errors (MAE and RMSE) by the purely on-the-edge learning algorithm presented in [32] that was numerically simulated on the same dataset.

Here, the “GS inputs” method is expected to perform better in short-term predictions given the pre-optimization of the input space based on the one-step ahead prediction performance, while the “PCA inputs” method is expected to perform better in longer-term predictions given its capability to extract physics-informed features. Numerical simulations were performed in Python 3 using a self-developed code. Although the LSTM cells in the ensemble were set to run in parallel, parallel computing was not implemented in the code used here.

## 5. Implementation and Results

This section presents the numerical results for the implementation of the algorithm on the drop tower dataset. First, the input space is constructed based on the source domain data. After, step ahead predictions are conducted in the target domain, and performance quantified.

### 5.1. Input Space Construction

**Figure 6:**
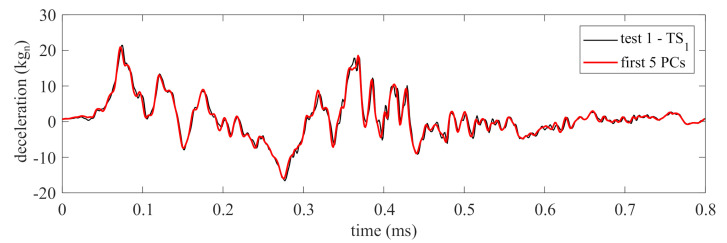
Comparison of the source domain measurements and its reconstruction using the first five PCs.

PCA was conducted on the source domain using a delayed observation matrix with the number of columns m=40, obtained heuristically. The first five PCs were selected as the basis signals of the measurements based on the capability of these PCs to reconstruct 95% of the original signal, and the 95% threshold was selected arbitrarily. Figure 6 compares the source domain signal “test 1—TS${}_{1}$” versus the reconstructed signal using the PCs (“first 5 PCs”). The figure shows that the reconstructed signal closely resembles the original signal.

**Figure 7:**
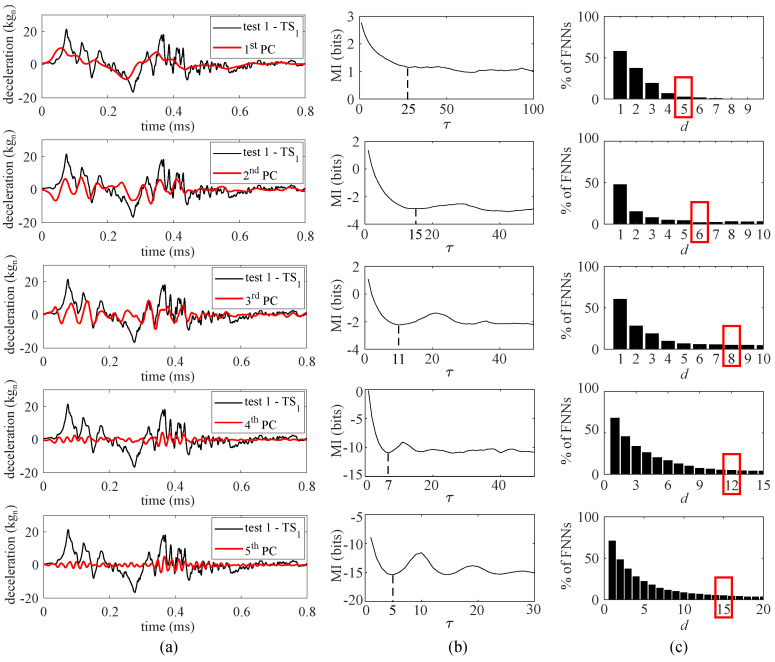
Selection of input space hyper-parameters: (**a**) PCA decompositions of the source domain measurements; (**b**) selection of τ based on MI analysis for each PC decomposition; and (**c**) selection of *d* based on percentage of FNNs for each PC decomposition.

Because measurements were decomposed using five PCs, five feature extractors were trained, each with an input space designed based on the associated PC signal. The reconstruction of the time series based on the individual PCs is visualized in Figure 7a. It can be observed that the PC time series moves from a slow-varying process with the first PC to a faster varying process with the fifth PC, thus exhibiting distinct temporal behaviors. The input space hyper-parameters τ and *d* were obtained based on the reconstructed time series (Figure 6, “first 5 PCs”). The value for τ is taken as the first local minima of the MI test value (Figure 7b) [40]. The dimension *d* used for each feature extractor is taken at the FNN percentage threshold of 5% (Figure 7c).

The five sets of input space hyper-parameters (τ and *d*) selected by using the “PCA inputs” method, along with those using the “GS inputs” method, are listed in Table 1. A comparison of the hyper-parameters selected by the two strategies shows that the “GS inputs” method yields LSTM cells focusing on short timescales, with the maximum time delay taken as τ=14, while the “PCA inputs” method selected a maximum of τ=25. In addition, the embedding dimensions of the PCA-based input parameters are generally smaller than those of the GS-based input parameters, which may indicate less averaging of past data points in the former method.

### 5.2. Time Series Prediction

Based on the results from PCA, five RNNs with LSTM cells, with their hidden states h of size equal to twice their corresponding input dimension *d*, were trained (Figure 3) for two epochs using a batch size of 10 and a learning rate of 0.005. The training was repeated for the RNNs based on the GS inputs. The LSTM weights of the RNNs were then taken as the feature extractor weights for prediction in the target domain. Using the input space hyper-parameters and the trained feature extractors, real-time learning and prediction was implemented (Figure 1) using a learning rate of 0.01 for both the attention layer and the linear neuron, and a fine-tuning rate of 0.001 for the feature extractors.

**Figure 8:**
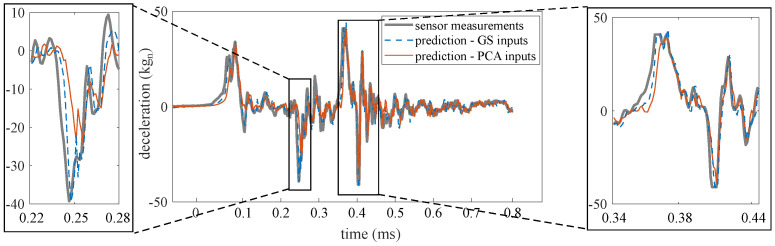
One-step ahead prediction, “PCA inputs” versus “GS inputs”.

**Figure 9:**
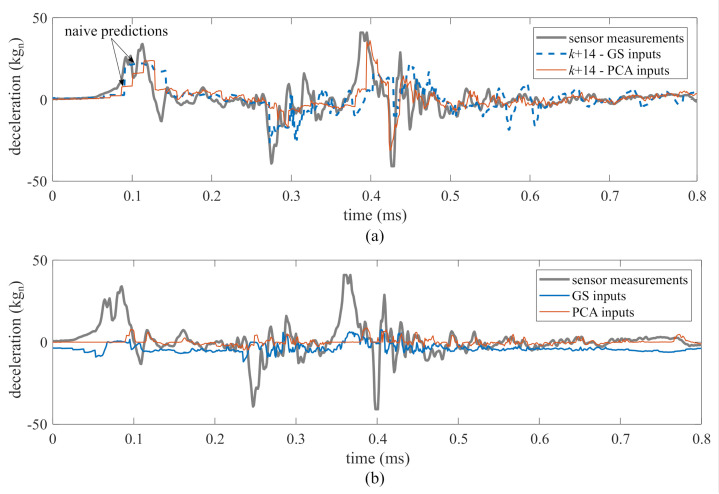
Prediction performance for 14 steps ahead, “PCA inputs” versus “GS inputs”: (**a**) prediction; and (**b**) scaled typical feature extracted by the LSTMs using τ=11 under both methodes.

Prediction performance under both methods was assessed for one- to 36-step ahead prediction using the performance metrics discussed in Section 4.2. Prediction horizons beyond 36 steps do not exhibit good performance overall. To allow for visual interpretation of the performance metrics Figure 8 and Figure 9 illustrates the prediction time series for two typical prediction horizons. Figure 8 shows a typical one-step ahead prediction by the ensemble of LSTM cells on test 1, TS${}_{2}$, under both the “PCA inputs” and “GS inputs” methods. Both methods yielded predictions that show good agreement with the experimental data, with “GS inputs” outperforming “PCA inputs”, as hypothesized, with an MAE of 1.2 kg${}_{n}$ by “GS inputs” compared to an MAE of 2.4 kg${}_{n}$ by “PCA inputs”. The under-performance of “PCA inputs” is noticeable in the zoomed portions of the time series. Figure 9a shows a similar comparison, but for a 14-step ahead prediction. Here, the “PCA inputs” method clearly outperforms the “GS inputs” method, with the respective MAEs of 3.5 kg${}_{n}$ and 4.3 kg${}_{n}$. The “PCA inputs” method also yields predictions with less chattering. Naive predictions can be observed as flat predictive trajectories under both methods at the beginning of the excitation. Figure 9b compares the time history of one of the features extracted from the LSTM cell that used τ=11 in its input space under both methods. The features were scaled linearly for better visual comparison. Features extracted from the LSTM cell with “PCA inputs” exhibit a good correspondence with the measured time series, where some of the peaks and valleys of the features match the corresponding peaks and valleys in the measurements, unlike the features extracted using the “GS inputs” method. The quantification of similarity between the time series features and measurements using the DTW metric will be discussed later in this section.

**Figure 10:**
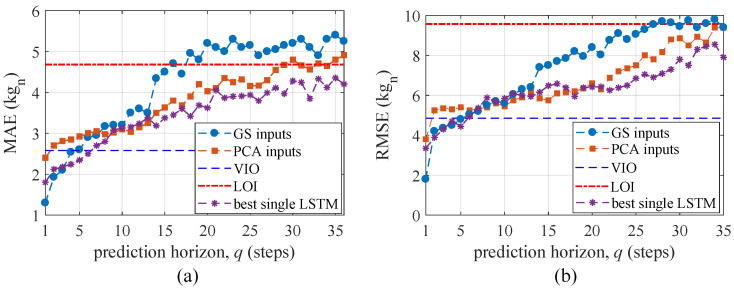
Error metrics (**a**) MAE; and (**b**) RMSE.

The average MAE and RMSE obtained over tests 1–5 from TS${}_{2}$ are plotted in Figure 10 for prediction horizons up to 36 steps ahead and benchmarked against variable input observer (VIO) method, a line of indifference (LOI) and the best single LSTM. The VIO line indicates the performance obtained in prior work [32] for one-step ahead prediction only, using a purely on-the-edge machine learning technique. Note that the VIO was only validated for one-step ahead predictions. Results are also compared against those obtained from the best single LSTM under each *q* to study the gain in performance from using the ensemble of LSTMs.

As expected, the algorithm based on “GS inputs” outperformed that based on “PCA inputs” over short prediction horizons since the input space of the former was optimized for one-step ahead prediction. The algorithm based on “GS inputs” outperforms the VIO over the prediction horizons of one to four steps, which constitutes a significant improvement. The “PCA-inputs” method only outperforms the VIO over the one-step ahead prediction. Overall, the LSTM-based predictor is a net improvement with respect to the VIO algorithm when comparing one-step ahead predictions. Importantly, the “PCA inputs” method quickly outperforms the “GS inputs” method and exhibits more stable performance over longer horizons, yielding better performance for approximately q≥6 steps.

A line of indifference (LOI) is defined as the limit where the prediction is not better than taking the mean value of the time series. Using the number of steps *q* when the algorithm surpasses the LOI as a limit on prediction horizon performance suggests that the “GS inputs”-based algorithm is useful at predicting up to 16 steps ahead based on the MAE and 27 steps ahead based on the RMSE, while the “PCA inputs” method is useful at predicting up to 29 steps ahead based on the MAE and 34 steps ahead based on the RMSE, given the LOI. The better performance of the “GS inputs” method can be attributed to the optimization of the hyper-parameters using the one-step ahead prediction performance target that inherently emphasizes short-term predictions. Results show that, compared with the best single LSTM, the “PCA inputs” method performs similarly yet with a slight underperformance, in particular over large prediction horizons, showing that the ensemble method can appropriately weigh information from the useful LSTMs. Note that using the best LSTM for prediction is not practical and is only useful for benchmarking purposes, given that no error metric is available over the prediction horizon enabling identification of the best LSTM.

**Figure 11:**
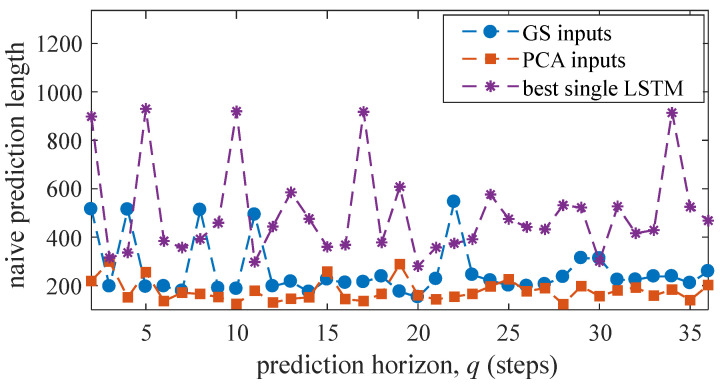
Mean naive prediction length for all tests.

Figure 11 plots the naive prediction length averaged over all five tests under both input space methods. Although “GS inputs” exhibited better performance over short horizons based on the MAE and RMSE metrics, it tends to behave more naively over these short horizons. This may to some extent explain the performance improvement of “GS inputs” relative to “PCA inputs”, because naive predictions tend to give a good approximation of the trajectory ($x_{k}\approx x_{k+q}$). It can also be observed that the output of the best single LSTM has a high degree of naivety, indicating this LSTM failed at extracting appropriate features on its own. Results show that the naive prediction length under “PCA inputs” is relatively constant, without spikes.

**Figure 12:**
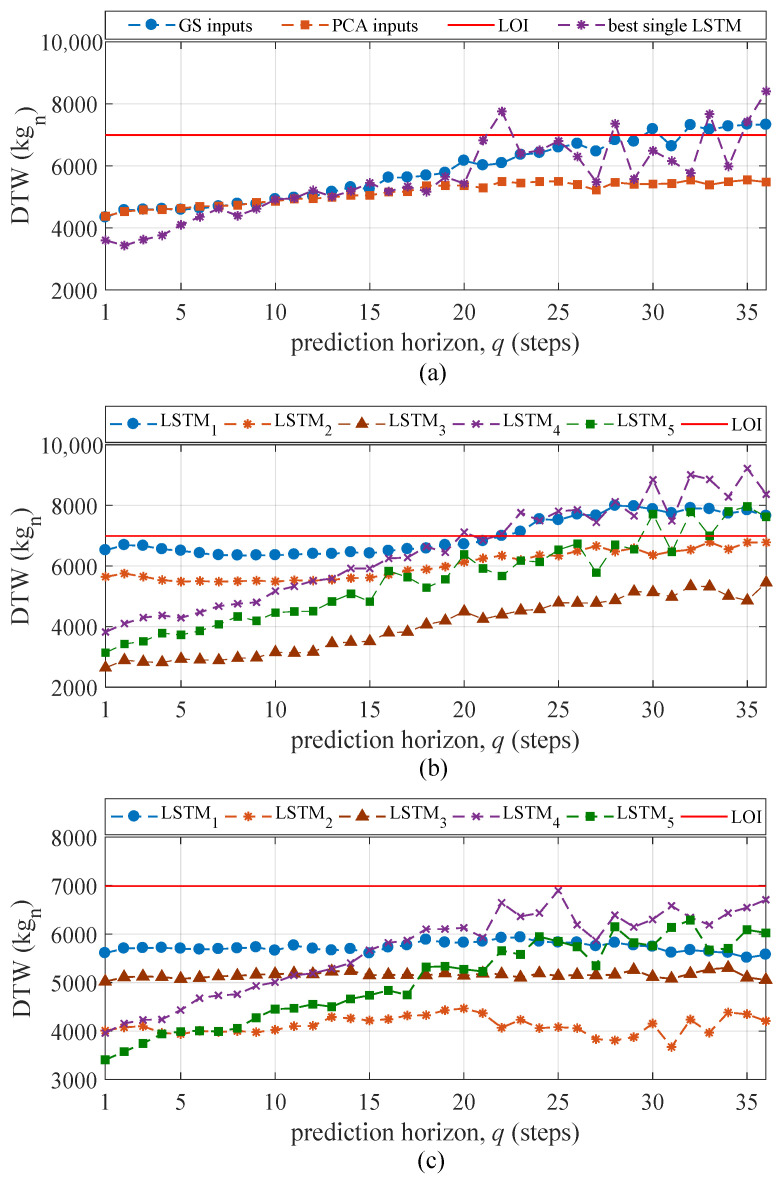
(**a**) Mean DTW over all LSTMs and five tests; (**b**) mean DTW of individual LSTMs over all five tests—“GS inputs”; (**c**) mean DTW of individual LSTMs over all five tests—“PCA inputs”.

Figure 12a plots the mean DTW of all the feature extractors over all five tests along with the LOI. For prediction horizons q≤12, the DTW metric for the extracted features is similar between the “GS inputs” and “PCA inputs” methods. The best single LSTM underperforms the “PCA inputs” method for long prediction horizons (q≥20), which confirms the LSTM’s inefficacy in extracting temporal features without the ensemble. The DTW for “GS inputs” increases rapidly and crosses the LOI at approximately q=30 while the mean value of DTW stays approximately at the same level. The DTW for the “PCA inputs” method remains low compared to that of the “GS inputs” method and does not cross the LOI, indicating that the PCA-based input space enabled a better reconstruction of features that are topologically similar to the signal (see Figure 9b). Figure 12b,c plot the DTW for individual feature extractors (LSTM cells) averaged over all five tests for “GS inputs” and “PCA inputs”, respectively. It can be observed that the DTWs for LSTM cells 1–3 in “GS inputs” extract better features in terms of similarity to the measurements, which yielded better performance over short prediction horizons. However, the performance starts to degrade with the increase of the prediction horizon, with three LSTMs surpassing the LOI for large prediction horizons. The performance of “PCA inputs” remains under the LOI over all prediction horizons.

## 6. Discussion

The presented deep learning algorithm was constructed for HRSHM applications, with the promise that the incorporation of physics could enhance feature extraction performance, thus yielding better predictions. The physical information stemmed from the PCA that extracted data structure from the training data, followed by the extraction of essential dynamics characteristics (τ, *d*) through the multi-rate sampler. Results showed that the proposed “PCA inputs” method did extract better features when compared against the “GS inputs” method, because (1) as exhibited in Figure 9b, the selected hidden state under “PCA inputs” followed changes in the time series significantly more closely; (2) the naive component (Figure 11) of “PCA inputs” was stable through all of the prediction horizons; (3) the DTW metric (Figure 12) showed a level of similarity between the sequence extracted by each LSTM and the source domain over all prediction horizon, which was not the case for “GS inputs”; (4) there was a net improvement in step ahead prediction capabilities (Figure 10), with “PCA inputs” surpassing the LOI for up to 29 steps ahead under the MAE metric and 35 steps ahead under the RMSE metric, compared against “GS input” surpassing the LOI for up to 15 and 27 steps ahead under the MAE and RMSE metrics, respectively; and (5) the use of an ensemble is critical in extracting useful temporal features compared to using a (hypothetical) best single LSTM under each prediction horizon. It follows that, with this particular data set, the incorporation of physics through the multi-sampler that aims at extracting essential dynamics out of different temporal characteristics obtained from PCA was a suitable approach and yielded important improvement in predictive performance.

In terms of HRSHM applicability, it is important for the computing time of the algorithm to remain under the prediction horizon for real-time implementation. The presented algorithm, coded in Python, had an average computing time of 25 μs. This would govern what should be the minimum data sampling rate, which here was 1 μs in the given dataset. It must be remarked, however, that the execution of the feature extractors was not conducted in parallel in the code, and that such parallel implementation combined with the use of more efficient coding techniques is expected to significantly decrease computing time. This is also true for implementations of the algorithm on hardware, such as on a field-programmable gate arrays (FPGA) or micro-controller. It should also be noted that while LSTM networks were used in this paper to validate the multi-rate sampling method, other time-series modeling algorithms could have been considered. An example is the gated recurrent unit (GRU) network that has exhibited promise in recent literature [47]. The study of different time-series modeling algorithms is left for future work.

It is also important to note that step ahead predictions are difficult to use directly into a decision making process, and that these predictions would need to be combined with other algorithms to empower the feedback system at the expense of additional computing time. One of the most straightforward applications of step ahead predictions to HRSHM could be the computation of a binary state damage/undamaged by comparing drifts in prediction errors, or by simply leveraging step ahead predictions in state estimation algorithms to augment the available computing time for real-time applications. More advanced implementations may use the step ahead prediction in parallel with an adaptive physical representation to obtain actionable information based on physics. This could be, for example, the extraction of local stiffness values in order to be capable of localizing and quantifying damage. Along with appropriate prognostic and remaining useful life models, this could constitute a powerful tool in making high-rate real-time decisions.

## 7. Conclusions

In this work, a deep learning algorithm for real-time prediction of high-rate sensor data was presented. The algorithm consisted of an ensemble of short-sequence long short-term memory (LSTM) cells that are concurrently trained. The main novelty was the use of a multi-rate sampler designed to individually select the input space of each LSTM based on local dynamics extracted using the embedding theorem, therefore providing improved step-ahead prediction accuracy and longer prediction horizons by extracting more representative time series features. This construction of the input space was conducted by first decomposing the signal using principal component analysis (PCA), and second selecting the individual delay vectors that extracted the essential dynamics of each principal component based on the embedding theorem.

The performance of the algorithm was evaluated on experimental high-rate data gathered from accelerated drop tower tests, consisting of time series measurements acquired from two accelerometers over five consecutive tests. The data from one sensor over a single test (TS${}_{1}$—test 1) was used to construct the input space and pre-train feature extractors. The data from the second sensor was used as the target domain to evaluate predictive capabilities. Performance was assessed in terms of mean absolute error (MAE), root mean square error (RMSE), and naivety of the prediction, as well as feature-signal similarities quantified through dynamic time warping (DTW). Performance of the proposed method (“PCA inputs”) was benchmarked against a more heuristic selection of the input space tailored to one-step ahead performance termed grid search inputs (“GS inputs”).

Results from the numerical simulations showed that, on this particular data set, the “PCA inputs” method outperformed the “GS inputs” method for prediction horizons beyond 6 steps in terms of MAE and RMSE. The “PCA inputs” also showed stability in terms of prediction naivety over the entire 36 steps-ahead prediction horizon. A study of the DTW metric showed superior performance of the “PCA inputs” in terms of extracting features that shared similarities with the sensor measurements. The enhanced predictive performance of the “PCA inputs” method can be attributed to this capability of extracting useful features from the highly non-stationary time series. The average computing time per discrete time step during the online prediction task was 25 μs, below the maximum useful of 29 steps, equivalent to 29 μs, under the MAE metric, thus demonstrating the promise of the algorithm at high-rate structural health monitoring (HRSHM) applications. Moreover, the comparison of the ensemble of LSTMs with a single LSTM showed the stability of the ensemble in terms of naive behavior of the predictions. It should be remarked that the computing speed of the algorithm could be improved by enabling parallel processing of the LSTM cells, and through hardware implementation.

Overall, this study shows the promise of a data-based technique at quickly learning a non-stationary time series and conducting predictions based on limited training datasets. While its performance could differ if applied to different time series dynamics, it was shown that it is possible to conduct adequate predictions of complex dynamics in the sub-millisecond range. It should also be noted that, to fully empower HRSHM, such predictive capability must be mapped to decisions through the extraction of actionable information. Future research is also required to investigate domain adaptation methods for situations involving source and target domains that differ significantly.

## Figures and Tables

**Table 1:** Input space hyper-parameters selected using the “GS inputs” and “PCA inputs” techniques.

	GS Inputs		PCA Inputs	
LSTM	τ	*d*	τ	*d*
1	14	8	25	5
2	11	10	15	6
3	8	12	11	8
4	5	14	7	12
5	4	15	5	15

## Abbreviations

The following abbreviations are used in this manuscript:

ms	milliseconds
HRSHM	High-Rate Structural Health Monitoring
RNN	Recurrent Neural Network
LSTM	Long Short-Term Memory
PCA	Principal Component Analysis
PC	Principal Component
MHz	Megahertz
kg${}_{n}$	Kilo g${}_{n}$ (gravitational acceleration)
MAE	Mean Absolute Error
RMSE	Root Mean Square Error
DTW	dynamic Time Warping
LOI	Line Of Indifference
VIO	Variable Input Observer
GRU	Gated Recurrent Units
